# Circulating Thrombospondin‐4‐Positive Fibroblasts Might be a Useful Marker for Diagnosis of Gastric Cancer

**DOI:** 10.1002/cam4.71700

**Published:** 2026-03-24

**Authors:** Koji Maruo, Masakazu Yashiro, Tomoya Sano, Daiki Imanishi, Takashi Sakuma, Yasuhiro Fukui, Hinano Nishikubo, Kyoka Kawabata, Rika Aoyama, Yurie Yamamoto, Fan Canfeng, Gen Tsujio, Tomohiro Sera, Atsushi Sugimoto, Kenji Kuroda, Hiroaki Kasashima, Kiyoshi Maeda

**Affiliations:** ^1^ Department of Gastroenterological Surgery Osaka Metropolitan University Graduate School of Medicine Japan; ^2^ Molecular Oncology and Therapeutics Osaka Metropolitan University Graduate School of Medicine Japan; ^3^ Cancer Center for Translational Research Osaka Metropolitan University Graduate School of Medicine Japan

**Keywords:** cancer‐associated fibroblast, early gastric cancer, Thrombospondin‐4, tumor marker

## Abstract

**Background:**

Cancer‐associated fibroblasts (CAFs) have been reported to be tumor‐specific cells. We have recently reported that thrombospondin‐4 (THBS4) expression is exclusive to CAFs. This study aimed to clarify whether the identification of circulating CAFs (cir‐CAFs) by THBS4 is detectable in the blood of gastric cancer (GC) patients, and whether cir‐CAFs are useful for the screening test of GC.

**Materials and Methods:**

CAFs and normal fibroblasts (NFs) were respectively established from 17 GC specimens. A total of 24 healthy volunteers and 77 GC patients were enrolled. Flow cytometric analysis was performed using anti‐THBS4 antibody. The sensitivity and specificity of THBS4‐positive cir‐CAFs for detection of GC were calculated.

**Results:**

THBS4+ cells showed ovoid‐like cells and expressed THBS4. THBS4^+^ expression was significantly (*p* = 0.014) higher on CAFs than NFs. GC patients had a significantly (*p* = 0.0323) higher average number of THBS4‐positive cir‐CAFs than healthy volunteers: 212 cells versus 6.4 cells. The ROC curve indicated that 27 THBS4‐positive cells per 10,000 blood cells was an adequate cutoff for GC diagnosis. The sensitivity and specificity of cir‐CAFs were 76.6% and 100%, respectively. In contrast, the sensitivities of CEA and CA19‐9 were only 22.1% and 9.1%, respectively. The sensitivity of cir‐CAFs was high even for Stage I GC, at 73.5%, while the sensitivity of CEA and CA19‐9 was low at 14.7% and 0%, respectively.

**Conclusion:**

THBS4‐positive cir‐CAFs are detectable in the blood of GC patients. The cir‐CAFs might be a useful tumor marker in a GC screening test, especially for early‐stage GC.

## 1 Introduction

Gastric cancer (GC) is the 5th leading cause of death and the 3rd most common cancer in Japan [1]. Despite the recent development of new GC therapies, including both surgical treatments and anticancer drugs, the 5‐year survival rate of GC remains low at around 70% [2, 3]. One of the reasons for the poor prognosis of GC might be the low diagnosis rate of GC at the early stage. Endoscopy has been a powerful tool for diagnosing GC, but endoscopy is not adequate for universal screening of GC. While the measurement of blood tumor markers is noninvasive and suitable for screening tests, most tumor markers are not useful for early detection of GC because of their low sensitivity and specificity [4]. Development of a novel tumor marker with high sensitivity and specificity is necessary to increase the diagnostic rate of GC in the early stage.

Cancer‐associated fibroblasts (CAFs) are characteristic stromal cells that are specifically recognized in tumor microenvironments [5]. It has been demonstrated that CAFs arise from various normal cell types, including fibroblasts, adipocytes, mesenchymal stem cells (MSCs), and pericytes [6, 7]. We previously reported that CAFs are derived from bone marrow MSCs via CXCL1 signaling from cancer cells [8]. These findings suggest that the identification of circulating CAFs (cir‐CAFs) in the blood may be a promising screening method to diagnose GC in the early stage. Although many markers derived from cancer cell components have been studied, no useful diagnostic markers for early‐stage GC have been identified.

Thrombospondin 4 (THBS4) is one of the five THBS family members and functions as a glycoprotein [9, 10]. Recently, it has been reported that THBS4 is expressed in CAFs in GC and breast cancer [11, 12]. Since to date there has been no report analyzing the suitability of cir‐CAFs as a tumor marker, this study aimed to clarify whether cir‐CAFs expressing THBS4 are detectable in the blood, and if so, whether cir‐CAFs would be a useful diagnostic marker for GC, especially in the early stage.

Recently, we have published the next 3 papers showing the significance of THBS4 of CAF in gastric cancer. Förster S. et al. first reported that THBS4 is a novel stromal molecule of diffuse‐type gastric adenocarcinomas by transcriptome‐wide analysis of gastric cancer and is frequently expressed by CAFs [13]. Kuroda K. et al. also reported that THBS4 was expressed on CAFs in the gastric cancer microenvironment and was a useful prognostic indicator for gastric cancer patients [12]. Tsujio G. et al. currently reported that THBS4 derived from CAFs might downregulate HER2 (*ERBB2*) expression in GC cells [14]. Also, THBS4 has been reported to be overexpressed on CAFs in several types of cancer such as GC, gallbladder cancer, and breast cancer, and plays an important role in tumor remodeling and angiogenesis [11, 15, 16, 17]. In this study, we aimed to clarify whether cir‐CAFs expressing THBS4 are detectable in the blood, and whether cir‐CAFs would be a useful diagnostic marker for GC.

## 2 Materials and Methods

### 2.1 Patients and Healthy Volunteers

The criteria for selecting GC patients was patients who were histologically diagnosed as gastric adenocarcinoma and underwent gastrectomy at our hospital from December 2022 to February 2024. A total of 77 GC patients were enrolled. The criteria for selecting healthy volunteers was clinically cancer‐free individuals aged between 25 and 60 years old. A total of 24 clinically cancer‐free persons were enrolled as healthy volunteers. This study was approved by the Ethics Committee of our hospital (reference nos. 924 and 3159). Informed consent was obtained in writing from all patients, and the study was conducted according to the principles of the Declaration of Helsinki.

### 2.2 Fibroblasts Established From GC Specimens

Seventeen paired cultures of CAFs and normal fibroblasts (NFs) were respectively established from 17 GC specimens of GC patients who underwent gastrectomy. CAFs and NFs from GC specimens were established as previously reported [12]. CAFs were obtained from the tumoral gastric wall tissue, and NFs were obtained from the noncancerous gastric wall tissues. These tissues were excised under aseptic conditions and minced with forceps and scissors. The primary culture was initiated as follows: each gastric specimen was excised under aseptic conditions and minced with forceps and scissors. The culture medium consisted of DMEM (Wako, Osaka, Japan) supplemented with 10% fetal bovine serum (FBS; Nichirei Biosciences Inc., Tokyo), 100 IU/mL penicillin (Wako), 100 mg/mL streptomycin (Wako), and 0.5 mM sodium pyruvate (Wako). Cells were cultured in 21% O2 at 37°C. Cells were then serially passaged every 4–7 days. These 17 paired cultures of CAFs and NFs were used from passages 2 to 4.

### 2.3 FACScan Analysis of CAFs and NFs

A total of 2 × 10^5^ CAFs or NFs were incubated with BD Cytofix Fixation buffer for 10 min at 4°C, followed by incubation with BD Phosflow Perm Buffer III for 20 min at 4°C. The sample was stained with anti‐THBS4 antibody (ab263898, 1:700; Abcam, Waltham, MA) conjugated with anti‐rabbit IgG labeled by Alexa Fluor^R^488 (ab150077, 1:2000; Abcam, Cambridge, UK) or an IgG isotype control conjugated with anti‐rabbit IgG (Alexa Fluor^R^488) (ab150077, 1:2000; Abcam, Cambridge, UK). Flow cytometric analysis was performed using a BD LSR II FACScan flow cytometer (Becton Dickinson, San Jose, CA).

### 2.4 Sample Preparation for Spike Test

From each of 2 healthy volunteers, an 8 mL aliquot of peripheral blood was collected and mixed with 1 × 10^4^ CAFs. Mononuclear cells were then isolated from the peripheral blood by Ficoll–Paque density centrifugation (Becton Dickinson) at 1500 *g* for 15 min. The mononuclear cell fraction was incubated with BD Cytofix Fixation buffer for 10 min at 4°C, and then incubated with BD PhosflowTM Perm Buffer III for 20 min at 4°C. The sample was then stained with anti‐THBS4 antibody (ab263898, 1:700; Abcam, Waltham, MA) conjugated with anti‐rabbit IgG labeled by Alexa Fluor^R^488 (ab150077, 1:2000; Abcam, Cambridge, UK). Flow cytometry analysis was performed on a BD LSR II FACScan flow cytometer (Becton Dickinson).

### 2.5 FACScan Analysis of Blood Samples From Patients and Healthy Volunteers

Preparation of peripheral blood from GC patients was performed as follows. Eight milliliters of peripheral blood was collected from each of the 77 GC patients and 24 healthy volunteers. Each mononuclear cells fraction of the peripheral blood was enriched by Ficol by 1500 *g* for 15 min. Flow cytometry analysis was performed in the same manner as for the spike test.

### 2.6 Cell Sorting Using FACScan and Immunohistochemistry

Cells were sorted by using the BD FACS Aria II (Becton Dickinson), and gating was done using the BD FACSDiva software (Becton Dickinson). Cell sorting was performed with a 100 μm nozzle size and sorted directly into 12 mL tubes containing 10 mL of staining media in order to minimize cellular stress. Cells (3000–20,000) of each population of interest were sorted. The THBS4^+^ cells sorted by FACS in the patient's peripheral blood were evaluated by Hematoxylin and Eosin (H&E) staining and THBS4^+^ staining.

### 2.7 CEA/CA19‐9 Levels

The levels of CEA/CA19‐9 of GC patients were measured in blood samples collected at the preoperative visit or on the morning of surgery, and were obtained from the medical records. The CEA/CA19‐9 levels of health controls were measured in the morning. Serum CEA/CA19‐9 levels were measured using an Alinity i system (Abbott Laboratories, Green Oaks, IL).

### 2.8 Statistical Analysis

Statistical analyses were performed using the R statistical software (R Foundation for Statistical Computing, Vienna, Austria) for Mac OS X (version 3.5.2). Significance tests were conducted using the chi‐square test or Fisher's exact probability test, and *t*‐test. A *p value* < 0.05 indicated a statistically significant difference.

## 3 Results

### 3.1 THBS4 Expression Level on CAFs and NFs

**FIGURE 1:**
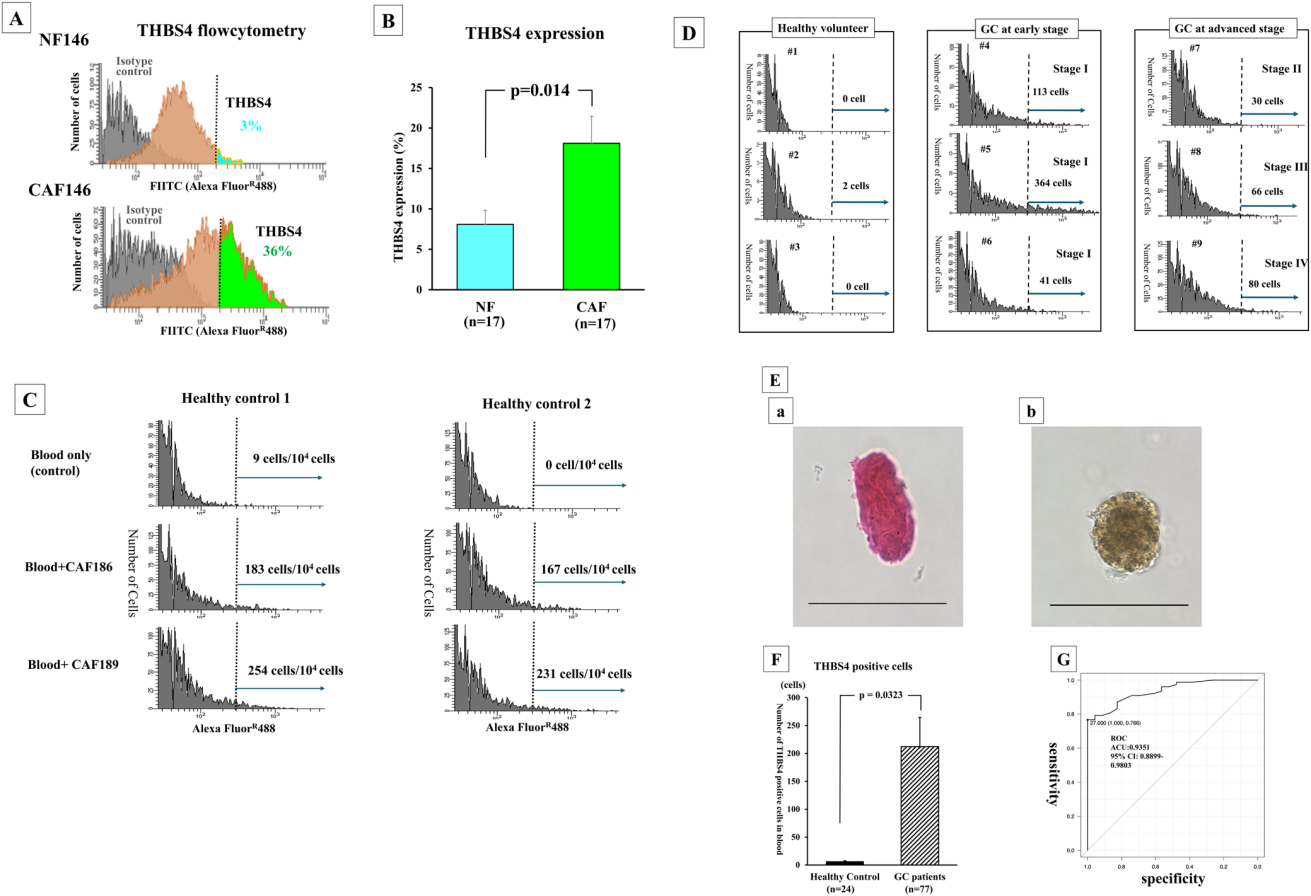
Flow cytometric analysis. (A) THBS4 expression level on CAF146 and NF146. (B) THBS4 expression on CAFs was significantly (*p* = 0.014) higher than that on NF in 17 paired CAFs and NFs. (C) Cutoff value of THBS4‐positive fluorescence (Alexa Fluor488) was determined to be 3 × 10^2^. (D) shows representative FACScan images of 3 healthy volunteers, 3 GC patients of early stage, and 3 GC patients of advanced stage. (E) The THBS4^+^ cells sorted by FACS in patient's peripheral blood were evaluated by H&E staining and THBS4 staining. Bar; 50 μm (F) THBS4‐positive cells in blood of 24 healthy volunteers and 77 GC patients. (G) shows ROC curve of THBS4‐positive cells for the sensitivity and specificity to GC.

Figure 1A shows a representative FACScan image. CAF146 more highly expressed THBS4 (36%) compared to NF146 (3%). THBS4 expression on CAFs (18.1% ± 3.5%) was significantly (*p* = 0.014) higher than that on NF (8.1% ± 1.8%) in the 17 paired cultures of CAFs and NFs (Figure 1B).

### 3.2 Determination of THBS4‐Positive Fluorescence and the Cutoff Value of THBS4‐Positive Cells in Blood

Next, 10,000 cultured CAFs were spiked into each 2 mL sample of peripheral blood from healthy volunteers. The results of the FACScan analysis demonstrated that the cutoff value of THBS4‐positive fluorescence was 3 × 10^2^ (Figure 1C). FACS Flow cytometric analysis was performed using blood samples from healthy volunteers (*n* = 24), GCs at Stage I (*n* = 34), GCs at Stage II (*n* = 10), GCs at Stage III (*n* = 12), and GCs at Stage IV (*n* = 21). Figure 1D shows representative FACScan images of 3 healthy volunteers, 3 GC patients in early stage, and 3 GC patients in advanced stage. The cells sorted by FACS in a patient's peripheral blood were stained by H&E and immunohistochemistry staining. The cells sorted by FACS showed ovoid‐like cells (Figure 1Ea) and THBS4‐positive cells (Figure 1Eb), which suggested that FACS analysis detected the cir‐CAF cells in peripheral blood.

The number of THBS4‐positive cells, as cir‐CAFs, in the samples of GC patients (*n* = 77) was 212 ± 52.4, versus only 6.4 ± 1.3 in healthy control samples (*n* = 24). GC patients had significantly (*p* = 0.0323) more THBS4‐positive cells than healthy controls (Figure 1F). Figure 1G shows the ROC curve for the number of THBS4‐positive cells in the blood of healthy controls and GC patients. The cutoff value of THBS4‐positive cells was determined to be 27 cells in 10,000 blood cells.

### 3.3 Sensitivity and Specificity of THBS4+ Cells, CEA, and CA19‐9 to GC

When the cutoff value of THBS4‐positive cells was > 27 cells, the sensitivity and specificity of THBS4‐positive cells were 76.6% and 100%. In contrast, CEA and CA19‐9 of the same samples showed that the sensitivity of CEA was 22.1% and the specificity of CEA was 100%, and the sensitivity of CA19‐9 was 9.1% and the specificity of CA19‐9 was 95.8% (Table 1). The sensitivity of GC at each stage remained as high as 73.5%, while the sensitivities of CEA and CA19‐9 were as low as 14.7% and 0%, respectively (Table 2).

**TABLE 1:** Sensitivity and specificity of THBS4^+^ cells, CEA, and CA19‐9 to GC.

Marker		Gastric cancer	Control (healthy volunteer)
		*n* = 77	*n* = 24
THBS4^+^ cells			
	≥ 27 cells	59	0
	< 27 cells	18	2
	Sensitivity/Specificity	76.6%/100%	
CEA			
	≥ 5.0 ng/ml	17	0
	> 5.0 ng/ml	60	24
	Sensitivity/Specificity	22.1%/100%	
CA19‐9			
	≥ 37.0 U/ml	7	1
	< 37.0 U/ml	70	23
	Sensitivity/Specificity	9.1%/95.8%	

**TABLE 2:** Sensitivity and specificity of THBS4^+^ cells, CEA, and CA19‐9 to GC at each stage.

Markers		Gastric cancer		Control
		Stage I	Stage II, III, and IV	(healthy volunteer)
		*n* = 34	*n* = 43	*n* = 24
THBS4^+^ cell	≥ 27 cells	25	35	0
	< 27 cells	9	8	24
	Sensitivity/Specificity	73.5%/100%	81.4%/100%	
CEA	≥ 5.0 ng/ml	5	12	0
	> 5.0 ng/ml	29	31	24
	Sensitivity/Specificity	14.7%/100%	27.9%/100%	
CA19‐9	≥ 37.0 U/ml	0	7	1
	< 37.0 U/ml	34	36	23
	Sensitivity/Specificity	0%/95.8%	16.3%/95.8%	

### 3.4 Significance of THBS4‐Positive Cells in the Clinicopathological Characteristics of GC Patients

We divided the GC patients into a cir‐CAFs positive group (> 27 THBS4‐cells; *n* = 59 patients) and a cir‐CAFs negative group (*n* = 18). The correlation between clinicopathologic factors and cir‐CAFs cases is shown in Table 3. There was a significant correlation between THBS4^+^ cases and tumor depth (*p* = 0.0277). cir‐CAFs cases showed a nonsignificant trend of correlation with CEA (*p* = 0.0597). In contrast, no correlation was observed for age, lymph node metastasis, distant metastasis, cy, vascular invasion, or CA19‐9.

**TABLE 3:** Correlation between THBS4^+^ cells and clinicopathologic factors of GC.

	cir‐CAFs positive (≥ 27)	cir‐CAFs negative (< 27)	*p*
	*n* = 59	*n* = 18	
Age			
< 65 (*n* = 16)	10 (62.5%)	6 (37.5%)	0.184
≥ 65 (*n* = 61)	49 (80.3%)	12 (19.7%)	
Differentiation			
Undiff. (*n* = 40)	33 (82.5%)	7 (17.5%)	0.202
Diffi. (*n* = 37)	26 (70.3%)	11 (29.7%)	
Tumor depth			
T1 (*n* = 30)	19 (63.3%)	11 (36.7%)	0.0277
T234 (*n* = 47)	40 (85.1%)	7 (14.9%)	
Lymph node metastasis			
Negative (*n* = 38)	28 (73.7%)	10 (26.3%)	0.547
Positive (*n* = 39)	31 (79.5%)	8 (20.5%)	
Distant metastasis			
Positive (*n* = 20)	17 (85%)	3 (15%)	0.372
Negative (*n* = 57)	42 (73.7%)	15 (26.3%)	
cy			
Positive (*n* = 14)	11 (78.6%)	3 (21.4%)	1.00
Negative (*n* = 63)	48 (76.2%)	15 (23.8%)	
ly			
Positive (*n* = 25)	19 (76%)	6 (24%)	0.929
Negative (*n* = 52)	40 (76.9%)	12 (23.1%)	
v			
Positive (*n* = 22)	19 (86.4%)	3 (13.6%)	0.247
Negative (*n* = 55)	40 (72.7%)	15 (27.3%)	
CEA			
Positive (*n* = 17)	16 (94.1%)	1 (5.9%)	0.0597
Negative (*n* = 60)	43 (71.7%)	17 (28.3%)	
CA19‐9			
Positive (*n* = 7)	5 (71.4%)	2 (28.6%)	0.663
Negative (*n* = 70)	54 (77.1%)	16 (22.9%)	

## 4 Discussion

This is the first report of the detection of cir‐CAFs in the blood of GC patients using THBS4 as a marker for CAFs. It has previously been reported that CAFs specifically appear in cancer tissues and play an important role in the progression of GC [18].

Four kinds of molecules including THBS4, podoplanin, fibroblast activation protein (FAP), and α‐smooth muscle actin (αSMA) have been reported to be markers for fibroblasts. Kuroda K et al. reported that podoplanin and αSMA were immunohistochemically expressed on both CAFs and NFs in GC specimen [12]. In this study, transcriptome‐wide analysis indicated that *THBS4* mRNA level was higher in CAFs compared with NFs, while *FAP*, *podoplanin*, and *αSMA* levels showed no difference between CAFs and NFs (data not shown). These findings suggested that podoplanin, FAP, and αSMA were not specific to CAF; then we used THBS4 as a CAF‐specific marker in this study.

We previously reported that THBS4 was immunohistochemically stained at the cytoplasm and/or cell membrane of CAFs, but not cancer cells in GC specimen [12]. In this study, we found that THBS4 was highly expressed on cultured CAFs but not on cultured NFs by using 17 paired cultures of CAFs and NFs derived from GC tissues. Förster S et al. [13] and Kuroda K et al. [12] reported that THBS4 was expressed in CAFs but not that of NFs by western blot analysis or immunohistochemical analysis. In this study, flow cytometric analysis showed that THBS4 expression was highly found on CAFs but not on NFs (Figure 1A). Also, flow cytometric analysis of the peripheral blood indicated that THBS4‐positive cells were frequently found in blood from GC patients, but rarely found in blood from healthy volunteers. These findings suggested that peripheral blood cells with THBS4 expression were exclusive to CAF, and suggesting that THBS4 is a beneficial marker to detect cir‐CAFs in the blood.

To date, there has been no evidence that CAFs exist as circulating cells in the blood of GC patients. We therefore examined whether THBS4‐positive cir‐CAFs are detectable in the blood of GC patients. A total of 24 healthy volunteers and 77 GC patients were enrolled. Our analysis revealed that THBS4‐positive cir‐CAFs were more frequently recognized in GC patients than in healthy controls. Next, we examined whether cir‐CAFs are a useful marker for the diagnosis of GC, especially for early GC at Stage I. We found that the sensitivity and specificity of THBS4‐positive cir‐CAFs were 76.6% and 100%, respectively. Also, the sensitivity of cir‐CAFs remained as high as 73.5% in 34 patients with early‐stage GC. These findings suggested that cir‐CAFs with THBS4 expression might be a useful diagnostic marker for GC, particularly for early‐stage GC.

THBS4‐positivity of cir‐CAFs was significantly correlated with tumor depth. In contrast, no correlation was observed for age, sex, lymph node metastasis, distant metastasis, cy, vascular invasion, or CA19‐9. Förster S. et al. previously reported that THBS4 showed the strongest correlation to diffuse type [13], but there was no correlation in our data.

It has been reported that CAFs play an important role in the malignant development of GC [19, 20]. cir‐CAFs might be one of the prospective markers for the malignant potential of GC.

Carcinoembryonic antigen (CEA) and carbohydrate antigen 19–9 (CA19‐9) have been popular tumor markers in gastrointestinal tumors [21, 22]. In our analysis, CEA showed a trend of correlation with positivity for cir‐CAFs, which suggested that cir‐CAFs may be as or more effective than CEA as a marker of GC. The sensitivity of cir‐CAFs (76.6%) was higher than that of CEA (22.1%) or CA19‐9 (9.1%) in the same 77 patients. Also, the cir‐CAFs were found in 73.5% of the 34 patients with early‐stage GC, while CEA was detected in only 14.7% of the early GC patients. These findings suggested that cir‐CAFs with THBS4 expression might be a more promising marker for GC than other conventional markers, and may be suitable for use in a cancer screening test.

Because of the high specificity of cir‐CAFs exploring THBS4's as a diagnostic marker, the cir‐CAFs are useful as second screening tools for patients with positive tumor markers such as CEA and CA19‐9. A prospective study is necessary to put this into practical use. It also might be important to further expand the scope of research to examine whether THBS4‐positive cir‐CAFs might be useful to detect not only GC but also other types of cancer. Biliary tract cancer and pancreatic cancer are also accompanied by abundant CAFs in the tumor microenvironment [5]. cir‐CAFs may also be a useful marker for the early detection of these types of carcinomas.

Several limitations were acknowledged. There was variation in sample size between stages. Since this study was conducted at a single institution, regional, and racial bias cannot be denied. Multi‐institutional research will be necessary in future. A prospective study is also necessary to put this into practical use.

In conclusion, cir‐CAFs might be a promising tumor marker for diagnosing GC, especially GC at an early stage.

## Author Contributions

K.M. performed the experiments of this study, interpreted the data, and wrote the manuscript; M.Y. designed the experiments of this study, interpreted the data, and edited the manuscript; T.S., D.I., T.Sa., Y.F., H.N., K.Ka., R.A., Y.Y., F.C., G.T., T.Se., A.S., K.Ku., H.K. collected and analyzed patient data. Ki.M. supervised the work. All authors discussed the results and contributed to the final manuscript. All authors approved the manuscript.

## Conflicts of Interest

The authors declare no conflicts of interest.

## Data Availability

The data that support the findings of this study are available on request from the corresponding author. The data are not publicly available due to privacy or ethical restrictions.
